# State Medicaid Policy and LTC: A Multi-State Qualitative Study of Policy Influence on Caregiver Experiences

**DOI:** 10.1093/geroni/igab046.398

**Published:** 2021-12-17

**Authors:** Tonie Sadler, Kevin Yan, Daniel Brauner, Harold Pollack, R Tamara Konetzka

**Affiliations:** 1 The University of Chicago, Chicago, Illinois, United States; 2 Western Michigan University, Kalamazoo, Michigan, United States; 3 University of Chicago, Chicago, Illinois, United States

## Abstract

This study focuses on long-term care (LTC) state Medicaid policy and its impact on caregiver decisions and experiences. It examines respondents’ general knowledge of LTC state policies and services, challenges with navigating LTC policies and services, and decision-making pathways based on these factors. Using purposive sampling, 63 family caregivers across eight states participated in open-ended qualitative interviews (2019-2020) until thematic saturation was reached. Questions broadly examined caregivers’ experiences and decisions, focusing on decisions made around type of care setting and experiences with LTC state policy. States were selected to represent variation in Home and Community Based Service (HCBS) expenditures as a percentage of total Medicaid long-term services and support expenditures. While LTC policies and services vary significantly by state, we identified many parallels in caregiver experiences and perceptions across states, as respondents often lacked specific knowledge about LTC policies and services and how to access them. Overarching themes include LTC policy navigation challenges, distrust in state-funded LTC services and supports, and moral expectations of caregiving. To manage these challenges, caregivers employed coping strategies such utilizing informal support networks, hiring care coordination assistance, and “stretching things thin” to fill the policy and service gaps. Study findings highlight potential strategies to improve LTC services across states. There is a need to improve community trust with state services by employing transparent regulatory and evaluation procedures for LTC. Wider access to case management may improve communication and knowledge of available services to maximize benefit from HCBS expansions.

